# Distinguishing Drones from Birds in a UAV Searching Laser Scanner Based on Echo Depolarization Measurement

**DOI:** 10.3390/s21165597

**Published:** 2021-08-19

**Authors:** Jacek Wojtanowski, Marek Zygmunt, Tadeusz Drozd, Marcin Jakubaszek, Marek Życzkowski, Michał Muzal

**Affiliations:** Institute of Optoelectronics, Military University of Technology, 00908 Warsaw, Poland; marek.zygmunt@wat.edu.pl (M.Z.); tadeusz.drozd@wat.edu.pl (T.D.); marcin.jakubaszek@wat.edu.pl (M.J.); marek.zyczkowski@wat.edu.pl (M.Ż.); michal.muzal@wat.edu.pl (M.M.)

**Keywords:** UAV detection, drone detection, laser scanner, drone vs. bird discrimination, drone monitoring, anti-drone system

## Abstract

Widespread availability of drones is associated with many new fascinating possibilities, which were reserved in the past for few. Unfortunately, this technology also has many negative consequences related to illegal activities (surveillance, smuggling). For this reason, particularly sensitive areas should be equipped with sensors capable of detecting the presence of even miniature drones from as far away as possible. A few techniques currently exist in this field; however, all have significant drawbacks. This study addresses a novel approach for small (<5 kg) drones detection technique based on a laser scanning and a method to discriminate UAVs from birds. The latter challenge is fundamental in minimizing the false alarm rate in each drone monitoring equipment. The paper describes the developed sensor and its performance in terms of drone vs. bird discrimination. The idea is based on simple cross-polarization ratio analysis of the optical echo received as a result of laser backscattering on the detected object. The obtained experimental results show that the proposed method does not always guarantee 100 percent discrimination efficiency, but provides certain confidence level distribution. Nevertheless, due to the hardware simplicity, this approach seems to be a valuable addition to the developed anti-drone laser scanner.

## 1. Introduction

Drones’ capabilities to perform illegal observations or transport unauthorized objects, combined with low price and easy access, presents high risk and new security challenges to cope with [1,2,3,4,5,6,7]. The key technology associated with counteracting this issue is drone-oriented monitoring of airspace, especially above sensitive areas. In this context, the capability to distinguish between drones and birds is a vital factor to obtain low-false-alarm technology. There are a number of existing techniques, both passive and active, to detect drones remotely. The most common methods include the application of visual or thermal cameras [8,9,10], where typically long-range lenses are applied to analyze the image of the monitored sector. Unfortunately, taking into account the size of small UAVs and a large distance, even for high-resolution FPA sensors, a drone corresponds hardly to a few pixels. The identification is thus highly problematic. Increasing the focal length of the optics is a method to improve this situation, however at the same time is associated with the reduction of angular sector monitored, which is a significant drawback. As a result, the application of cameras for drone detection/identification cannot be used effectively on its own. It requires input from some assisting sensor providing possible direction to potentially suspicious objects. Radars constitute the second most common technology for drone detecting systems [11,12,13,14,15,16,17,18,19,20]. The assets of the corresponding methods are indisputable. The analysis of micro-Doppler shifts in the detected frequencies allows even to assess the number of UAVs rotors. However, the main problem results from extremely low radar cross-section of small drones located in large distances. Audio detection also has been proposed as an effective method for UAVs detection [21,22,23]. Flight of even the smallest drone cannot be realized noiselessly. The effectiveness of audio methods associated with analyzing acoustic spectra to find signatures of drones, strongly depends on the distance, background noise, and finally the completeness of the data base covering the a.m. signatures. Radio-frequency or Wi-Fi detection [24,25,26,27] both constitute another passive methods to detect UAVs which typically use these channels to communicate with an operator. Unfortunately, it is not always the case. Sometimes drones do not require any link, since their mission is pre-programmed. An excellent review of the current methods to detect and classify drones remotely is provided in [28], where the authors underline the problems with detecting small drones at large distances and distinguishing them from other flying objects. In order to increase the effectiveness of anti-drone sensors, the whole variety of advanced data analysis methods are implemented, including artificial network and deep learning techniques [26,29]. Depending on the complexity, different drone-detecting systems provide various levels of sensitivity, range, and effectiveness in localizing drones in space. Generally, it can be concluded however, that due to small cross-sections of many lightweight drones, most of the radar-based and image-based techniques seem to suffer from relatively short range. Concerning acoustic and radio/Wi-Fi methods, the main issue comes from the lack of drone positioning capabilities. 

Apart from just a drone detection, the demanding technical challenge of reliable discrimination capabilities—if drones and naturally occurring interfering factors (like birds), must be taken into account if a sensor is considered to work automatically. Nevertheless, a lot of effort has been allocated, especially in radar data processing in terms of such discrimination capabilities [14,17]. However, most of the currently operating anti-drone systems have to be assisted by an operator, who performs the final classification of the detected object and react accordingly. 

Our intention was to address the drone detection problem in a novel way to create a dedicated laser scanner, which additionally, in terms of detection and classification, could work partially unattended. Thus, it required to develop effective technique to distinguish between drones and birds, with minimum false alarm rate. To meet this challenge, our laser scanner was supplemented with depolarization analysis optical module. Another goal was to achieve detection maximum range at the required level of about 1 km, even regarding the small (c.a. 5 kg) UAVs. The approach to detect drones and identify them by the application of a laser scanner, to the best of our knowledge, was not reported before. According to the project limitations in revealing the details of scanning geometry, it is not discussed in this paper, which has been devoted solely to a “drones vs. birds” discrimination challenge.

## 2. Materials and Methods

Identification of surface type, its microscopic or macroscopic parameters, based on reflected light polarization state measurements have been reported many times [30,31,32,33,34,35,36,37,38,39,40,41]. Most of the research in this field however deals with laboratory methods based on full Stokes vector and Mueller matrix analysis. For example, in [30] authors analyze scatter polarization properties of selected man-made objects, using Mueller matrix imaging polarimeter technique. Variety of incoming polarization states and angles of incidence-reflection have been analyzed. The aim of this research was to determine classifiers which would easily enable to discriminate between man-made objects and natural background. In [32], the impact of surface micro-roughness and albedo on Mueller matrix components was studied. In [37], the authors prove that it is possible to discriminate between metallic and dielectric surfaces by analyzing the angular dependence of specularly scattered light. To achieve such discrimination however, one has to measure the polarization state of reflected light at numerous angles. Exploiting any useful phenomenon or property, lab methods are typically oriented at maximum possible precision, while measurements time and hardware requirements, are of less importance. On the other side, our work was primarily focused on a method, which despite its drawbacks and limitations, could give acceptable level of performance, but primarily could be easily implemented in a commercial device. Following this remark, we intentionally limited the measured depolarization effects, in order to reduce the developed device complexity, cost, and size.

**Figure 1:**
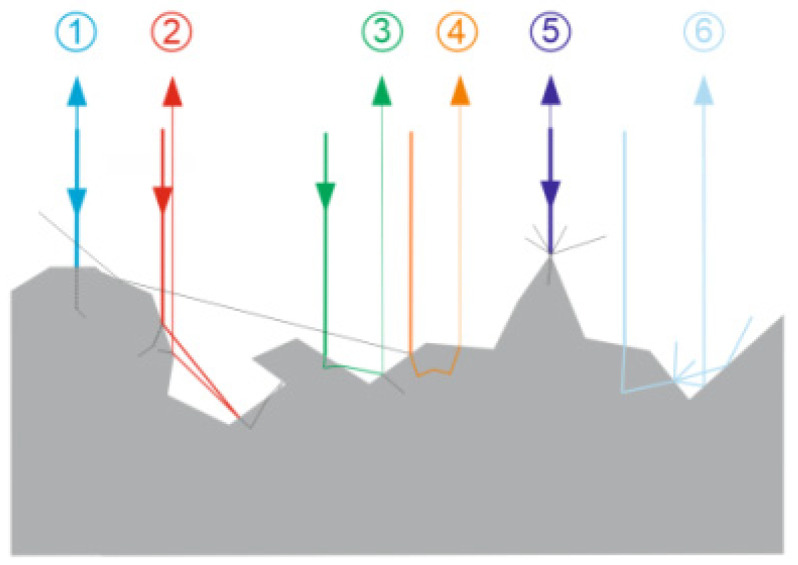
Heuristic presentation of a reflection of light from rough surface, as a combination of several physical mechanisms. Visualization of magnified rough surface micro-relief and six possible examples of how a ray of light can interact with it.

Drones are made of the whole variety of materials not only in terms of type, but also in terms of surface finish, ranging from well-polished to very rough. Nevertheless, polarized light, upon reflection (scattering) from any type of surface experiences changes in terms of the polarization state [35]. If mirror-like surface is considered, the phenomenon is described by well-known analytical Fresnel formulas. Reflected light keeps its high level of degree of polarization—*DoP* or polarizance [30,32], typically defined as

(1) $$DoP=\frac{\sqrt{Q+U+V}}{I},$$

where *I, Q*, *U*, *V* correspond to the Stokes vector **S** components typically addressed in

(2) $$S=\left[\begin{array}{c} I\\ Q\\ U\\ V \end{array}\right]=\left[\begin{array}{c} P_{x}+P_{y}\\ P_{x}-P_{y}\\ P_{45^{o}}-P_{135^{o}}\\ P_{right}-P_{left} \end{array}\right],$$

where *P_x_*—power of horizontal linear polarization component; *P_y_*—power of vertical linear polarization component; *P*_45_^o^—power of 45° oriented linear polarization component; *P*_135_^o^—power of 135° oriented linear polarization component; *P_right_*—power of right direction circular polarization component; and *P_left_*—power of left direction circular polarization component. For example, linearly polarized light, after reflection from metallic surface becomes elliptically polarized. The only case in which polarization state (every Stokes vector component) and *DoP* remains unchanged is normal incidence and back-reflection from a perfect mirror. On the other side, if reflection from rough surface or even more complicated one, like a biological tissue is considered, the rigorous mathematical description is complicated [34,36,38,40] and it is difficult to find any universal model capable of describing great variety of surface types. Nevertheless, reflection from a surface which cannot be treated as a smooth interface, becomes a complex process that can be heuristically compared to the combination of coexisting reflections, refractions, and scattering (Figure 1).

The relative contributions of the above-mentioned ingredients depend on material itself—its conductivity, refractive index, and surface irregularity sizes with respect to wavelength of light. For example, if metallic surface is considered, light will not penetrate into the volume of the object. Thus, factors numbered as 3, 4, and 6 in Figure 1 will not exist, however those numbered as 1, 2, and 5 will produce the main contributions. It is well known, that in general, each reflection/refraction leads to modification of Stokes vector (change of polarization state). Being a net effect of numerous acts of reflection/scattering on randomly oriented and shaped microscopic objects, reflection from rough surface corresponds to more or less depolarization, understood as the decrease of *DoP*. In other words, polarized incoming light, after reflection becomes only partially polarized or, in the extreme case, totally depolarized. The general rule states that the more complex surface micro-relief, the more depolarization it causes upon reflection. Quantitatively, a surface reflection induced polarization changes of a beam, are commonly described by the Mueller matrix **M** [31,32], which provides the full information how the Stokes vector transforms

(3) $$S_{out}=MS_{in},$$

where **S***_in_* and **S***_out_* refer to incident and reflected beam Stokes vector respectively. Thus, Mueller matrix provides complete description of the averaged effect of a surface on a polarization state transformation resulting from reflection of a beam from that surface. *DoP*, Stokes vector and Mueller matrix are very useful tools used to completely analyse beam polarization state and depolarization properties of surfaces. Unfortunately, their practical implementation in commercial laser scanner, where size and price are of high importance, could be problematic. For example, Stokes vector evaluation requires multiple power measurements of a beam with different configurations of polarizers. It is easily realizable in a lab, however considering the architecture of a laser scanner, would require applying several detection channels and a number of optical components. For this reason, our research was directed to verify measurement method that requires as little hardware as possible. Thus, instead of analysing the complete Stokes vector of the received optical echo signal, we investigated the simpler concept of measuring just cross-polarization ratio. It should be underlined, that such approach was the result of an acceptable compromise, since it requires to apply just two receiving paths. It is important to note, that we were not trying to build the best possible identification capability of the scanner, however just the classification potential. For example, it was not expected to be able to distinguish between different species of birds. It was also not intended to identify individual types of drones. The main goal of the developed sensor was just to distinguish drones from birds. 

**Figure 2:**
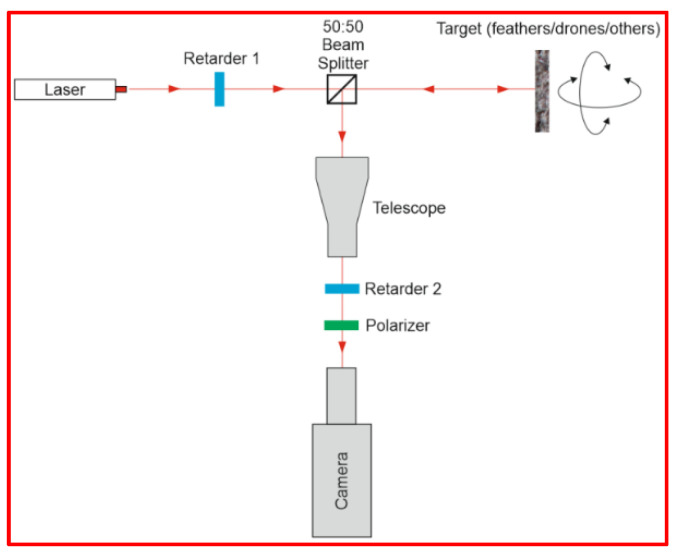
Laboratory setup for experimental search of optimum output/measured polarizations combination in terms of feathers vs. artificial surfaces discrimination.

Considering this challenge, it was reasonable to analyse the depolarization effects upon reflection from both UAVs surfaces and birds’ feathers. For this reason, laboratory experiments were carried out in the setup presented in Figure 2.

It allowed us to modify both output (Retarder 1) and measured (Retarder 2) polarization state. For each set of output/measured polarization combination, targets were swapped (feathers, drones, and other artificial materials that could potentially be an UAV surface). Also, the goal was to verify how the discrimination between feathers and artificial surfaces improves if more polarization settings in the detection channel are collected. In this way, we intended to find a compromise between the required complexity of the optical detection channel for the designed laser scanner and its acceptable level of performance. The main purpose of these measurements was to check if there is any specific output/measured polarization combination, in which feathers are significantly most differentiated from the rest of the targets. Measurements proved that the analysis of full Stokes vector will not result in proportional increase of system discrimination efficiency. The main reason comes from the fact, that if linearly polarized laser light is reflected from both a bird and a drone surface, the circular polarization in the scattered signal will be negligible. The main contributions are limited to linear polarization and depolarized light. 

**Figure 3:**
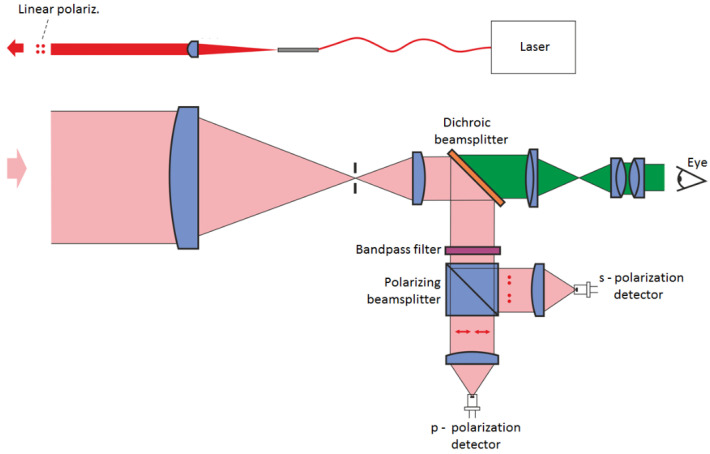
Optical configuration of the developed scanner (rotating mirror omitted).

Following the conclusions of laboratory experimental stage, the detection optical train for the developed anti-drone laser scanner was designed (Figure 3).

The concept relies on the standard polarization beamsplitter cube and two separate detectors—one responsible for s and the second for p polarization. Polarization axis direction of the beam (linearly polarized laser is applied), is alligned with the beamsplitter in such a way, that in case of no depolarization of optical echo, only s detector receives signal. On the other side, if echo is completely depolarized, one is tempted to say that both detectors would receive the equal power levels. Indeed, it would happen, however in case of the dichroic beamsplitter absence. In our system, dichroic beam splitter induces constant and deterministic depolarization factor. According to this scheme, in order to evaluate the discussed depolarization effects quantitatively, we introduced cross-polarization ratio *δ* defined as

(4) $$\delta =\frac{P_{p}}{P_{s}},$$

where *P_p_* and *P_s_* correspond to the power received by p and s detector respectively. Theoretically, *δ* can reach values in the range from zero to infinity, however taking into account partially depolarizing targets this domain is limited to (0 ÷ 1). Referring to Stokes formalism, one can easily arrive at the connection between *δ* and **S** components, namely

(5) $$\delta =\frac{I-Q}{I+Q}.$$

Being interested only to the first two Stokes vector components and taking into account Equation (3), it can be concluded that in terms of Muleller matrix description, our method is limited to half of this matrix, namely to components *M_ij_*, where *i* = {1, 2} and *j* = {1, 2, 3, 4}: $[\begin{array}{cccc} M_{11} & \;M_{12} & M_{13} & \;M_{14}\\ M_{21} & \;M_{22} & M_{23} & \;M_{24}\\ \;\;\;x\;x\; & \;x\;x\;\\ \;\;\;x\;x\; & \;x\;x\; \end{array}]$.

## 3. Results

### 3.1. Scanner Prototype Development

**Figure 4:**
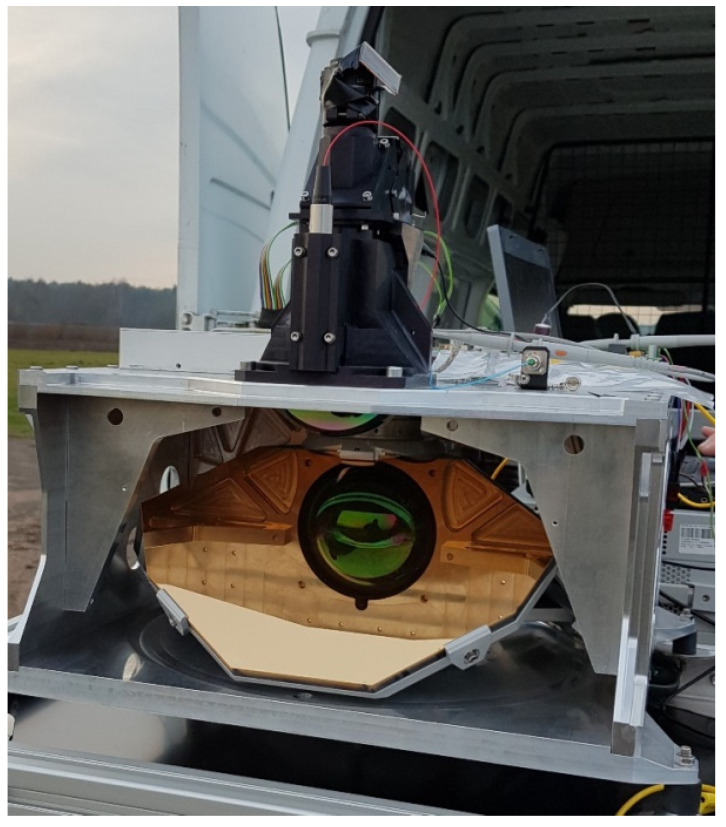
Photography of the constructed anti-drone laser scanner prototype.

The anti-drone scanner was designed and its prototype was created according to the discussed optical configuration (photography of the developed system in Figure 4). The platform is based on fiber pulsed laser operating at 1550 nm wavelength. Additionally, the dedicated software environment was developed. This prototype was used to perform a lot of experimental work both in a field and in a lab. It was our intention to collect parameters of the measured objects, not in the lab on some other apparatus, but applying directly the constructed device. In such approach, the results are affected by all the hardware constants specific for this instrument. Accordingly, we do not have to take care of the correction factors which would be the case upon transferring depolarization signatures from lab measurements to the scanner.

### 3.2. Experiments

**Figure 5:**
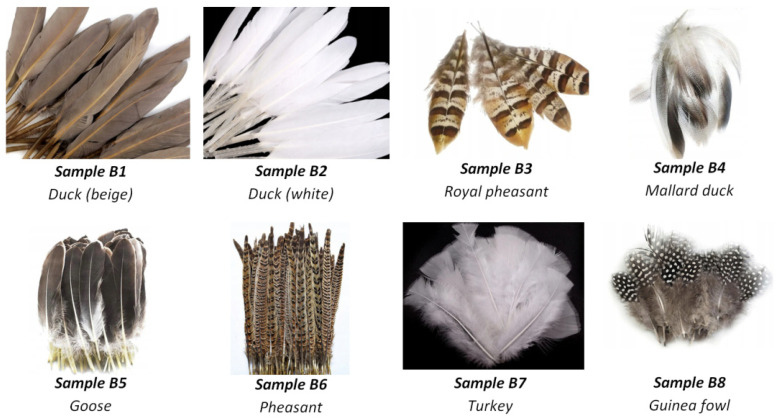
Samples representing birds.

**Figure 6:**
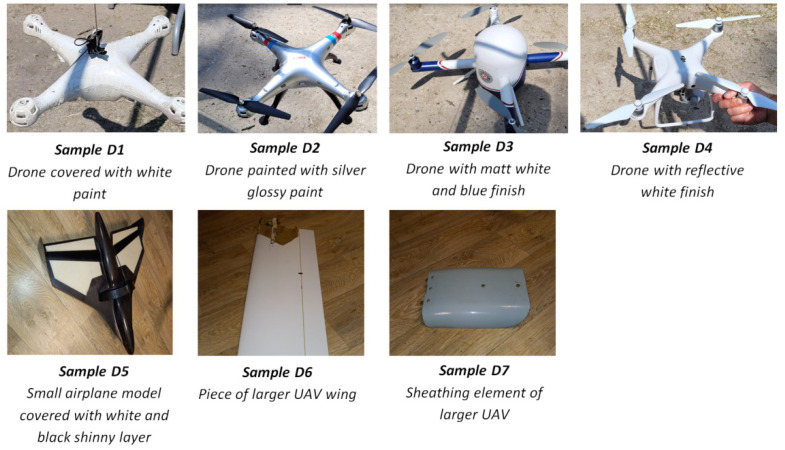
Samples representing drones.

First of all, the theoretical maximum detection ranges were confirmed in real conditions. Secondly, the depolarization signatures of drones were collected. So far, we have not arranged experiments with real birds flying in the air, however numerous targets prepared with a whole variety of feathers were fabricated. Each such target has been thoroughly investigated in terms of its cross-polarization ratio δ resulting from different orientations of its surface with respect to the laser beam direction. In effect, we collected the valuable statistics of depolarization signatures representing both drones and birds. In order to prepare the samples representing birds, we used the natural feathers of the species listed in Figure 5. On the other side, samples associated with UAV-type objects have been presented in Figure 6.

**Figure 7:**
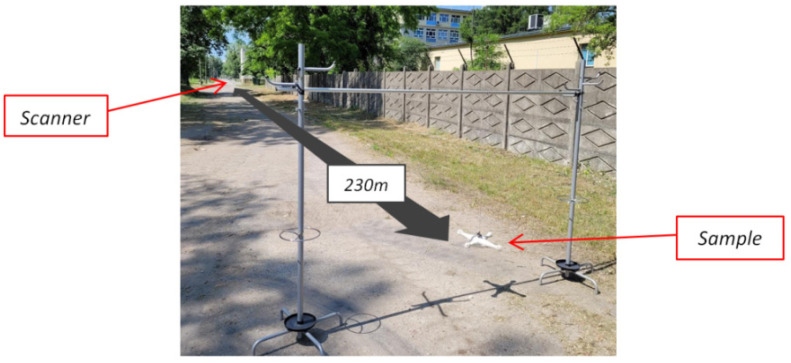
Photo of the experimental test site.

The experiments were performed in open terrain. Scanner was located in the distance of 230 m from the place where samples could be swapped (Figure 7). Such distance allowed to obtain 25 cm laser spot—sufficient size to average spatial irregularities effects of the samples. Additionally, the detection electronics could work at nominal settings, which would be not possible in shorter ranges, due to saturation.

**Figure 8:**
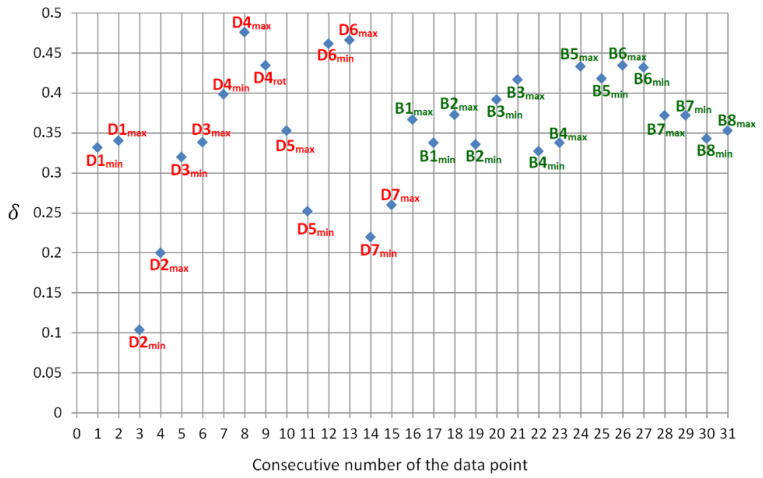
Results of cross-polarization ratio field measurements using the developed laser scanner module. Samples naming corresponds to Figure 5 and Figure 6 (red color—drones; green color—birds).

Each sample was measured in several angular orientations, in order to verify also the impact of this geometrical aspect. We changed the sample orientation in order to find the maximum and minimum level of the measured cross-polarization ratio. The results are summarized in Figure 8. It shows, for each measured sample, the obtained extreme values of *δ*.

For example, considering D7 it can be seen that the maximum measured cross-polarization ratio was about 0.27 and minimum observed δ dropped to 0.24. Any other angular orientations of this sample resulted it the values in between. Additionally, in case of D4 sample we got a single result while rotor was rotating (marked as D4_rot_). 

## 4. Discussion

**Figure 9:**
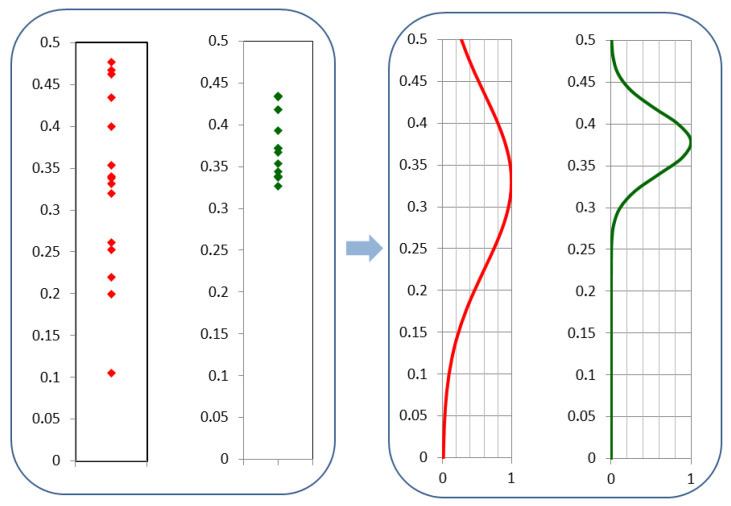
(**Left**) histograms of separated data sets corresponding to drones (in red) and birds (in green). (**Right**) normal distributions (normalized to unity) fitted to both histograms.

The visual inspection of the presented data allows to clearly distinguish the distributions features of the samples representing drones and those associated with birds. The former shows significantly broader range than the latter. Additionally, mean values seemed to be slightly shifted. Tempted to quantify these differences, we fitted normal distributions for both data groups (Figure 9).

It allowed us to asses both standard deviation σ and average (mean) values $\bar{\delta }$, which appeared to be:

(6) $$\{\begin{array}{c} \sigma_{\delta }\left(drones\right)=0.105\\ \bar{\delta }\left(drones\right)=0.33 \end{array}\;\;\;\;\;\;\;\;\;\;\;\;\;\;\;\;\;\;\;\;\;\;\;\;\;\;\{\begin{array}{c} \sigma_{\delta }\left(birds\right)=0.037\\ \bar{\delta }\left(birds\right)=0.38 \end{array}.$$

**Figure 10:**
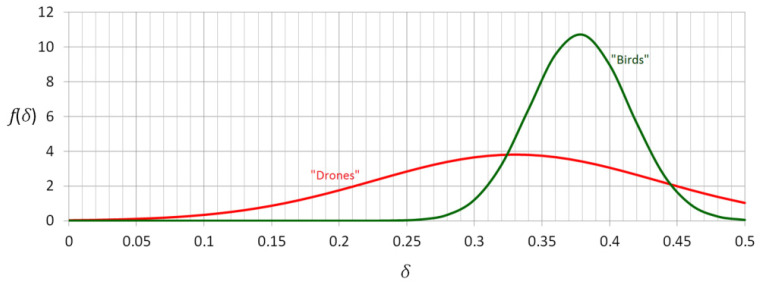
Probability density functions of *δ* (drones curve—in red, birds curve—in green).

It proved a large discrepancy especially in terms of standard deviations σ (0.105 vs. 0.037). The distribution associated with birds is significantly more tightly focused around its mean value. It corresponds to potential classification methodology. Namely, one can state that if the measured (by the developed scanner) cross depolarization ratio of unknown airborne object, is close to 0.38, at high probability it is a bird and at lower probability it is a drone. On the other side, if the obtained *δ* is far from 0.38, at high probability it is a drone and at lower probability it is a bird. Taking another example, if the measured value of *δ* goes below 0.27 level, there is very high probability, that drone was detected. Such approach is obviously not deterministic, since the obtained results revealed meaningful overlap between both data groups. Let us consider the unnormalized Gaussian distributions *f*(*δ*) associated with both data sets (Figure 10).

They can be interpreted as probability density distributions of obtaining the specific values of cross-polarization ratio both for drones and for birds. Namely, the probability *P* of obtaining *δ* in the range of any δ* ± (Δ/2) is equal to

(7) $$P\left(\delta^{*}\right)=\int_{\delta^{*}-\frac{\Delta }{2}}^{\delta^{*}+\frac{\Delta }{2}}\;\;f\left(\delta \right)d\delta .$$

In the discussed problem, we are more interested in the difference in probabilities of certain *δ* detection, if drones vs. birds are considered. Thus, let us implement the following classification probability factor

(8) $$\Delta P\left(\delta \right)=\frac{P_{drones}\left(\delta \right)-P_{birds}\left(\delta \right)}{P_{drones}\left(\delta \right)+P_{birds}\left(\delta \right)}.$$

Being the probability of obtaining any result (drone or bird), the denominator serves as a normalization component. Taking into account Equation (7) and the fact that integration ranges are identical, one obtains

(9) $$\Delta P\left(\delta \right)=\frac{f_{drones}\left(\delta \right)-f_{birds}\left(\delta \right)}{f_{drones}\left(\delta \right)+f_{birds}\left(\delta \right)}.$$

**Figure 11:**
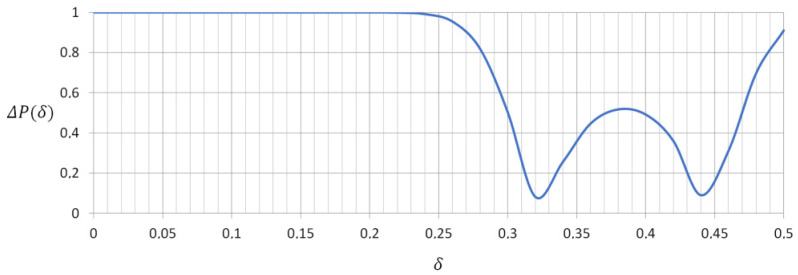
Drones vs. birds classification probability factor.

The plot of this function is presented in Figure 11. It can be interpreted as an indicator of the *δ* ranges, where the discrimination between drones and birds is efficient (Δ*P* ~ 1) and those *δ* ranges, where the a.m. discrimination potential is reduced (Δ*P* < 1).

Following the obtained Δ*P*(*δ*) curve, it can be concluded, that the proposed scanner solution and classification method efficiency depends on cross-polarization signature of a specific target. It may not be highly successful if the measured *δ* are in the vicinity of 0.32 and 0.44, which simply results from the equality of probability density distributions *f*(*δ*) for drones and birds. Nevertheless, one can also spot broad *δ* ranges, where the classification potential is very promising (Δ*P* ~ 1).

**Figure 12:**
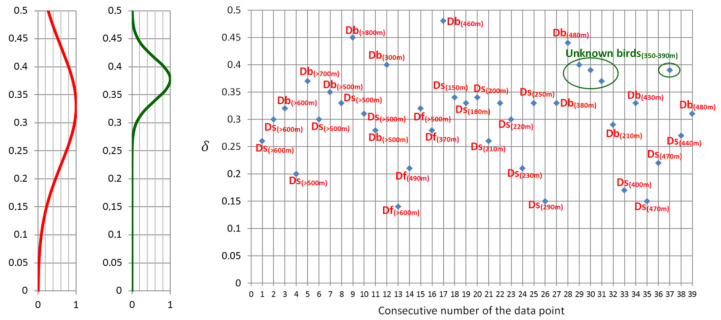
Comparison of the measured *δ* obtained for various distances (**right**) with two Gaussian curves corresponding to drones (red) and feathers (green) obtained in the static measurements (**left**). Db, Ds, Df – symbols associated with the specific drones models used in the experiments.

Additionally, it should be clarified that during the described experimental campaign the measurement distance was constant, so no impact of this parameter on the registered *δ* was analyzed intentionally. It results from the fact that the strength of the proposed depolarization signature lies in its independence of distance. Atmosphere propagation, especially on such short routes, does not induce significant change of polarization state. It is observed experimentally and also proved theoretically [42] that multiple Rayleigh scattering does not depolarize more than 1%. Nevertheless, in order to visualize experimental confirmation of our discrimination capability immunity to range, we overlaid the distributions obtained during the described campaign and the results obtained in the experiments devoted to the verification of our scanner range performance (Figure 12).

In such campaigns however, we did not have the opportunity to control the angular orientation of the targets precisely, so the presented data points correspond to single measurements. Anyway, one can easily notice that the rules deduced from static campaign are valid for dynamic experiments in various distances, too. The cross-polarization ratio *δ* associated with birds shows the mean value of 0.388 (0.38 for static campaign), while in case of drones (for all distances) it is 0.297 (0.33 for static campaign). Similarly, standard deviations of *δ* for drones and birds (0.09 and 0.01 respectively) shows the same discrepancy in distributions spread, as obtained in static experiments.

## 5. Conclusions

In this paper, a novel approach for drone detection and distinguishing them from birds was described. Unlike most current detection techniques in this field (radars, imaging, radio, and acoustic techniques) we proposed the application of a laser scanning. Regarding high angular resolution of this type of method, such approach enables to detect even the smallest drones at greater distances, which may become problematic for other techniques mentioned above. Additionally, similarly to radar technology, laser scanning provides precise location of the detected object in space, which is not possible in case of imaging, acoustic and radio frequency techniques. Going beyond just drone detection, we investigated the potential of reflected laser light depolarization signature as a discriminant between a drone and a bird. 

In order to verify the whole concept, we created a dedicated laser range-finding module, additionally equipped with cross-polarization ratio determination functionality. The module was used for numerous terrain experiments, where both the expected range and drone vs. bird discrimination capability were verified. The latter issue became the main subject of this paper. The experimental results and corresponding data analysis show, that cross-polarization ratio can be used for the a.m. discrimination, which leads to significant reduction of false alarms in the developed laser scanner. 

The proposed discrimination scheme is probabilistic in nature and its efficiency depends on the obtained cross-polarization ratio value. For some *δ* ranges, distinguishing a drone from a bird is straightforward, and for the others it is not. We identified these ranges, so the sensor algorithm is aware how likely a discrimination is successful. The obtained compromise is the cost of maximum simplicity of the implemented solution. The future tests, and especially the planned application of our scanner in real life border protection duty, will show if further improvements concerning the discussed discrimination efficiency are required. If so, in order to retrieve more Mueller matrix components associated with the detected object surface, additional channel or channels in detection unit will be implemented.

## Data Availability

Data is contained within the article.
